# One pot light assisted green synthesis, storage and antimicrobial activity of dextran stabilized silver nanoparticles

**DOI:** 10.1186/s12951-014-0053-5

**Published:** 2014-12-03

**Authors:** Muhammad Ajaz Hussain, Abdullah Shah, Ibrahim Jantan, Muhammad Nawaz Tahir, Muhammad Raza Shah, Riaz Ahmed, Syed Nasir Abbas Bukhari

**Affiliations:** Department of Chemistry, University of Sargodha, Sargodha, 40100 Pakistan; Drug and Herbal Research Centre, Faculty of Pharmacy, Universiti Kebangsaan Malaysia, Jalan Raja Muda Abdul Aziz, Kuala Lumpur, 50300 Malaysia; Institute of Inorganic and Analytical Chemistry, Johannes Guttenberg University of Mainz, Duesbergweg 10-14, Mainz, 55128 Germany; International Center for Chemical and Biological Sciences, University of Karachi, Karachi, 75270 Pakistan; Centre for Advanced Studies in Physics (CASP), GC University, Lahore, 54000 Pakistan

**Keywords:** Ag nanoparticles, Storage of nanoparticles, Diffused sun light, Antimicrobial activity

## Abstract

**Background:**

Green synthesis of nanomaterials finds the edge over chemical methods due to its environmental compatibility. Herein, we report green synthesis of silver nanoparticles (Ag NPs) mediated with dextran. Dextran was used as a stabilizer and capping agent to synthesize Ag NPs using silver nitrate (AgNO_3_) under diffused sunlight conditions.

**Results:**

UV–vis spectra of as synthesized Ag nanoparticles showed characteristic surface plasmon band in the range from ~405-452 nm. Scanning electron microscopy (SEM) and atomic force microscopy (AFM) studies showed spherical Ag NPs in the size regime of ~50-70 nm. Face centered cubic lattice of Ag NPs was confirmed by powder X-ray diffraction (PXRD). FT-IR spectroscopy confirmed that dextran not only acts as reducing agent but also functionalizes the surfaces of Ag NPs to make very stable dispersions. Moreover, on drying, the solution of dextran stabilized Ag NPs resulted in the formation of thin films which were found stable over months with no change in the plasmon band of pristine Ag NPs. The antimicrobial assay of the as synthesized Ag NPs showed remarkable activity.

**Conclusion:**

Being significantly active against microbes, the Ag NPs can be explored for antimicrobial medical devices.

## Introduction

Ag NPs have wide variety of applications, e.g., opto-electrical [1,2], microbiocidal [3], nanorobotics [4] and medicinal [5]. However, clustering of Ag NPs on storage and in physiological media [6-9] is a major limitation in their biomedical applications. Chemical methods for the synthesis of Ag NPs have harmful effects on environment as well as on human health [9]. Due to said reasons, nowadays, polysaccharides and polypeptides [10] have attracted the vigil eye of researchers for the biosynthesis of Ag NPs as they can act as reducing, capping and stabilizing agents [11-13]. Recently, polysaccharide based Ag NPs have been prepared by adding NaOH [14] but use of such corrosive reagent has harmful effects on environment as well as on human health. Therefore, it is promising to fabricate Ag NPs that could sustain themselves for longer period of time using environmentally benign molecules like biopolymers.

In this report, we have explored the green synthesis of Ag NPs using dextran as co-reducing as well as capping ligand without using any environmentally hostile ingredient like NaOH and NaBH_4_. Dextran was choice because it is cheaper, non-toxic, biocompatible, efficient reducing and self-capping agent, *in situ* stabilizer of nanoparticles and environment friendly. The as synthesized NPs can be stored within matrix of dextran in the form of thin films without changing the optical properties over months. Moreover, the as-prepared Ag NPs were tested as antimicrobial probes against *S. aureus* (ATCC 25923), *E. coli* (ATCC 25922)*, B. subtilis* (ATCC 6633)*, S. epidermidis* (ATCC 12228)*, P. aeruginosa* (ATCC 27853) and fungal strains *Actinomycetes and A. niger.*

### Experimental

#### Materials and measurements

Dextran (molar mass 40000) was obtained from Sigma Aldrich, Germany. AgNO_3_ (99.98%) from Merck, Germany was used as silver precursor. Deionized water was used for preparation of all solutions. UV–vis analyses were performed on UV-1700 PharmaSpec (Shimadzu, Japan). FT-IR spectra were recorded on IR Prestige-21 (Shimadzu, Japan). The samples (microtomes) were analyzed by SEM Plano (Wetzlar, Germany) using carbon stubs (carbon adhesive Leit-Tabs No. G 3347). The sizes and shapes of NPs were analyzed using AFM, Multimode, Nanoscope IIIa, Veeco, (California, USA) in tapping mode. Powder X-ray diffraction measurements were carried out (over a range of 5-100°, 2ϴ) on an Xpert Pro MPD, (PANalytical, The Netherlands) diffractometer equipped with monochromatic X-rays.

#### Sample preparation of AgNO_3_ and dextran

AgNO_3_ solutions (50, 75 and 100 mmol) were prepared by dissolving AgNO_3_ (0.85, 1.27 and 1.7 g, respectively) in deionized water. Concentrated solution of dextran was freshly prepared by dissolving dextran in deionized water (10 mL).

#### Synthesis of Ag NPs mediated by dextran

Freshly prepared AgNO_3_ (50 mmol, 2 mL) solution was added to the dextran solution (2 mL). The reaction mixture was exposed to diffused sunlight and color change was monitored over a period of 24 h by using UV–vis spectrophotometer. The same procedure was adopted for AgNO_3_ (75 and 100 mmol) solutions, respectively.

#### Thin film formation of dextran loaded with Ag NPs

Concentrated aq. solution of dextran loaded with Ag NPs (100 mmol) was kept in a petri dish for drying under air and stored.

#### Atomic force microscopy (AFM)

The samples were prepared by dissolving thin films in deionized water and dispersing them on freshly cleaved sheet of mica substrate. AFM images were recorded at ambient temperature and repeated with different concentrations of the samples.

#### Scanning electron microscope (SEM)

Surface of dextran thin films was analyzed by SEM to study geometry of embedded Ag NPs.

#### Antimicrobial activity of Ag NPs

The test organism *S. aureus* (ATCC 25923), *E. coli* (ATCC 25922)*, B. subtilis* (ATCC 6633)*, S. epidermidis* (ATCC 12228)*, P. aeruginosa* (ATCC 27853) and fungal strains *Actinomycetes and A. niger* were used for testing the antimicrobial activity of Ag NPs. The bacterial and fungal strains were procured from Microbiology Labs of Agriculture University, Faisalabad, Pakistan. Mueller Hinton Agar Media (Oxoid Ltd., England) was used for bacterial growth and Sabouraud Dextrose Agar (Hardy Diagnostics, USA) was used for fungal growth. Inoculums were prepared by transferring the microorganism culture in both tubes having 10 mL of respective broth media (Mueller Hinton broth for bacterial culture and Sabouraud Dextrose broth for fungal culture) and were inoculated for 24 h at 37°C for bacteria and 27-30°C for fungi. Seven days old culture of fungal strain was washed and suspended in normal saline solution. Then filtered through glass wool aseptically and incubated at 28°C. The tubes were shaken periodically to accelerate the growth of microorganisms. The turbidity of inoculums was adjusted by 0.5 Mc Farland Standard.

Antimicrobial assay of Ag NPs against different bacterial and fungal strains was conducted by disc diffusion method. *In vitro* antimicrobial activity was screened by using Mueller Hinton Agar plates for bacterial strains. Inoculum (0.1 mL) was spread uniformly on plates. Ag NPs solution was loaded on 6 mm discs of Whatman No. 1 filter paper. Loaded discs were placed on the surface of medium and plates were incubated for 24 h at 37°C. Pure DMSO (15–20 mL) loaded disc was used as negative control. At the end of incubation period, inhibition zones were measured in millimeters. These studies were performed in triplicate.

Similarly, antifungal activity of Ag NPs was screened on Sabouraud Dextrose Agar plates by using disc diffusion method and plates were incubated at 27-30°C for 36–48 h. After incubation period, zones of inhibition were measured.

## Results and Discussion

AgNO_3_ (50, 75 and 100 mmol) solutions mixed with concentrated dextran solution were colorless in the beginning but turned light brown after 10 min indicating the nucleation of Ag NPs. The color changed to ruby red after 60 min while chocolate red color was observed after 24 h indicating the completion of growth process.

**Figure 1:**
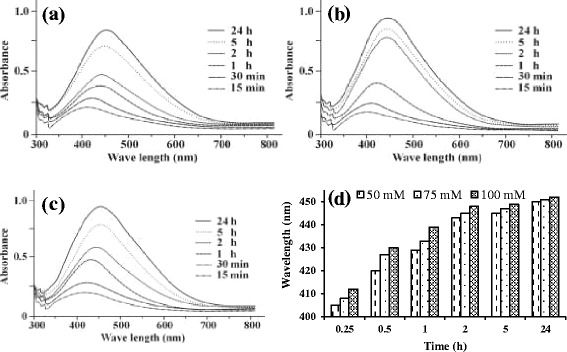
**UV–vis spectra of Ag NPs prepared in dextran: 50 (a), 75 (b) and 100 mmol (c) and graph showing effect of reaction time and concentration on absorbance (d).**

UV–vis absorption bands appeared ranging from ~405-450, 408–451 and 412–452 nm for nanoparticles synthesized using 50, 75 and 100 mmol dextran-AgNO_3_ solutions, respectively and the corresponding UV–vis spectra of dextran-Ag NPs are shown in Figure 1a,b and c. The all reactions were monitored for 24 h at different time intervals. The red shift was observed in UV–vis absorptions for dextran-Ag NPs by increasing reaction time. Increase in absorption coefficient was also observed by increasing the concentration of AgNO_3_ solution from 50–100 mmol. The increase in wavelength of absorption may be attributed to increase in size of Ag NPs. It is noteworthy that no absorption band was observed in the spectrum when sample was stored in dark. The reaction progressed on exposing the sample to diffused sunlight. Graphical representation of increase in absorption of Ag NPs solutions with increase in reaction time and AgNO_3_ concentration (50, 75 and 100 mmol) is depicted in Figure 1d.

**Figure 2:**
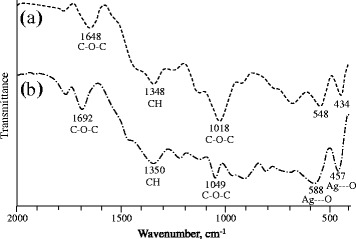
**FT-IR Spectra of dextran (a) and Ag NPs (50 mmol) loaded in dextran thin film (b).**

FT-IR (using pellet mixed with KBr) spectra of dextran and dextran-Ag NPs (50 mmol solution) were recorded to confirm interaction between dextran and Ag^+^ ions. Peaks at 434 and 548 cm^−1^ in pure dextran were shifted to 457 and 588 cm^−1^ in dextran-Ag NPs due to Ag---O excitation [15]. It is obvious from FT-IR spectra that there exist significant Van der Waal interactions between the chain of dextran and Ag NPs as all of the signals of dextran were shifted to somewhat higher positions (Figure 2).

**Figure 3:**
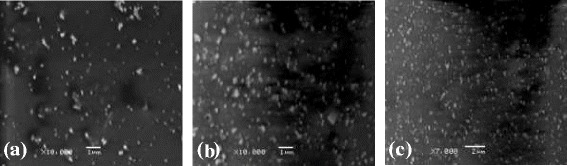
**SEM images of Ag NPs (50–70 nm) embedded in dextran thin films of 50 (a), 75 (b) and 100 mmol (c) AgNO**_**3**_ **solution.**

**Figure 4:**
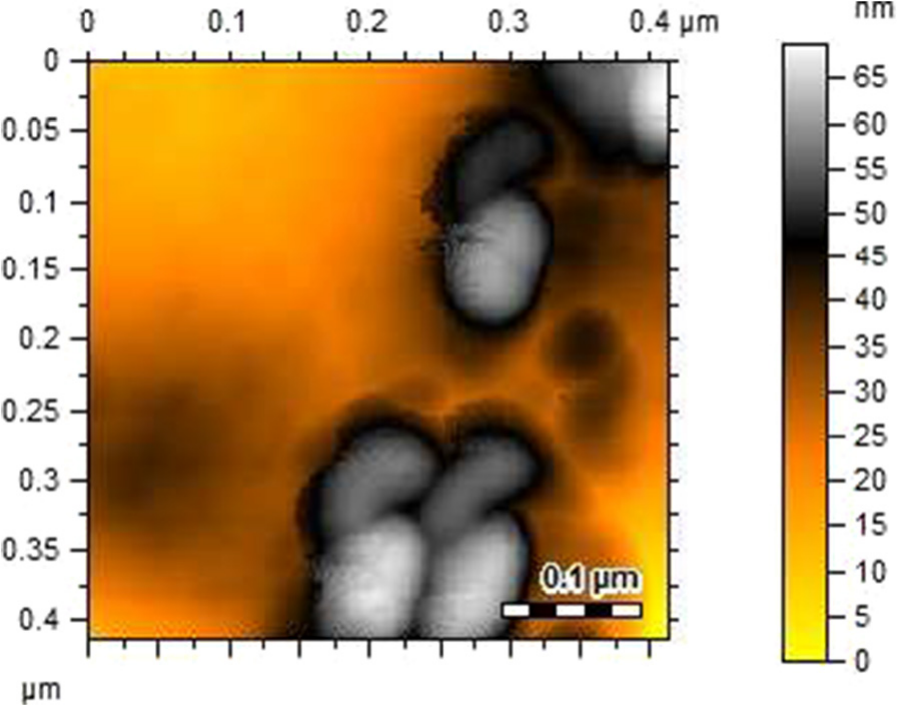
**AFM images of Ag NPs (50–70 nm) embedded in dextran thin films prepared from 100 mmol AgNO**_**3**_ **solution.**

Microtomes of dextran thin films loaded with Ag NPs observed by SEM showed spherical Ag NPs with uniform distribution (Figure 3). Dextran-Ag NPs film was dissolved in Milli-Q water and studied by AFM as well. The AFM images also witnessed the results of SEM that the NPs were found spherical (50–70 nm, Figure 4).

**Figure 5:**
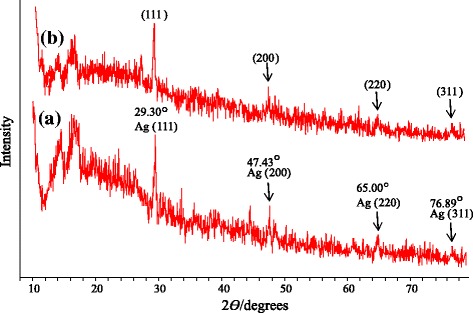
**PXRD Spectra of dextran-Ag NPs (100 mmol); (a) fresh sample and (b) recorded after one year storage.**

The crystalline nature of the as synthesized Ag NPs using dextran was confirmed via XRD analysis. As shown in Figure 5, there are four distinct reflections in the diffractogram at 29.3° (111), 47.43° (200), 65.05° (220), and 76.89° (311). These characteristics reflections show crystallographic planes of face centered cubic structure of the Ag NPs. Same sample was stored in the form of thin film for one year and PXRD was re-recorded to confirm the structural stability of the Ag NPs. Similarity of PXRD pattern in sample before and after one year (Figure 4) indicated that Ag NPs are quite stable on storage in thin film of dextran. In this way we could successfully avoid agglomeration of Ag NPs on storage in solid state. So, this novel method for long term storage of Ag NPs can be further exploited for potential biomedical applications and optoelectronic devices.

**Figure 6:**
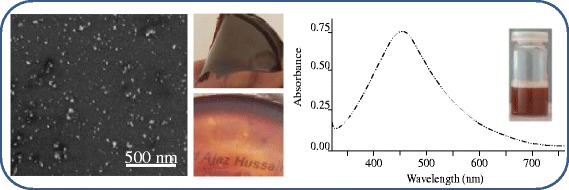
**SEM image and UV–vis spectrum of Ag NPs (100 mmol, 5 h reaction time) embedded in dextran thin films (foldable and see through) after one year storage; vial is indicating the color of stored thin films after dissolution in water.**

There was no difference in SEM images of the sample (100 mmol) before and after one year storage (Figure 6) in thin films. The synthesized thin films were foldable, ruby red in color and almost optically transparent as demonstrated by digital photograph (see Figure 6). Likewise, after one year storage of thin films under dark were re-dissolved in water. The UV–vis spectroscopic analysis of aqueous solution (100 mmol) of thin films (see Figure 6) after one year showed an absorption band centered at 446 nm. The concordant absorbance therefore indicated no change in size and morphology of the stored Ag NPs in thin films. So, the present method appeared highly efficient for the long term storage of Ag NPs in dextran thin films without agglomeration.

**Figure 7:**
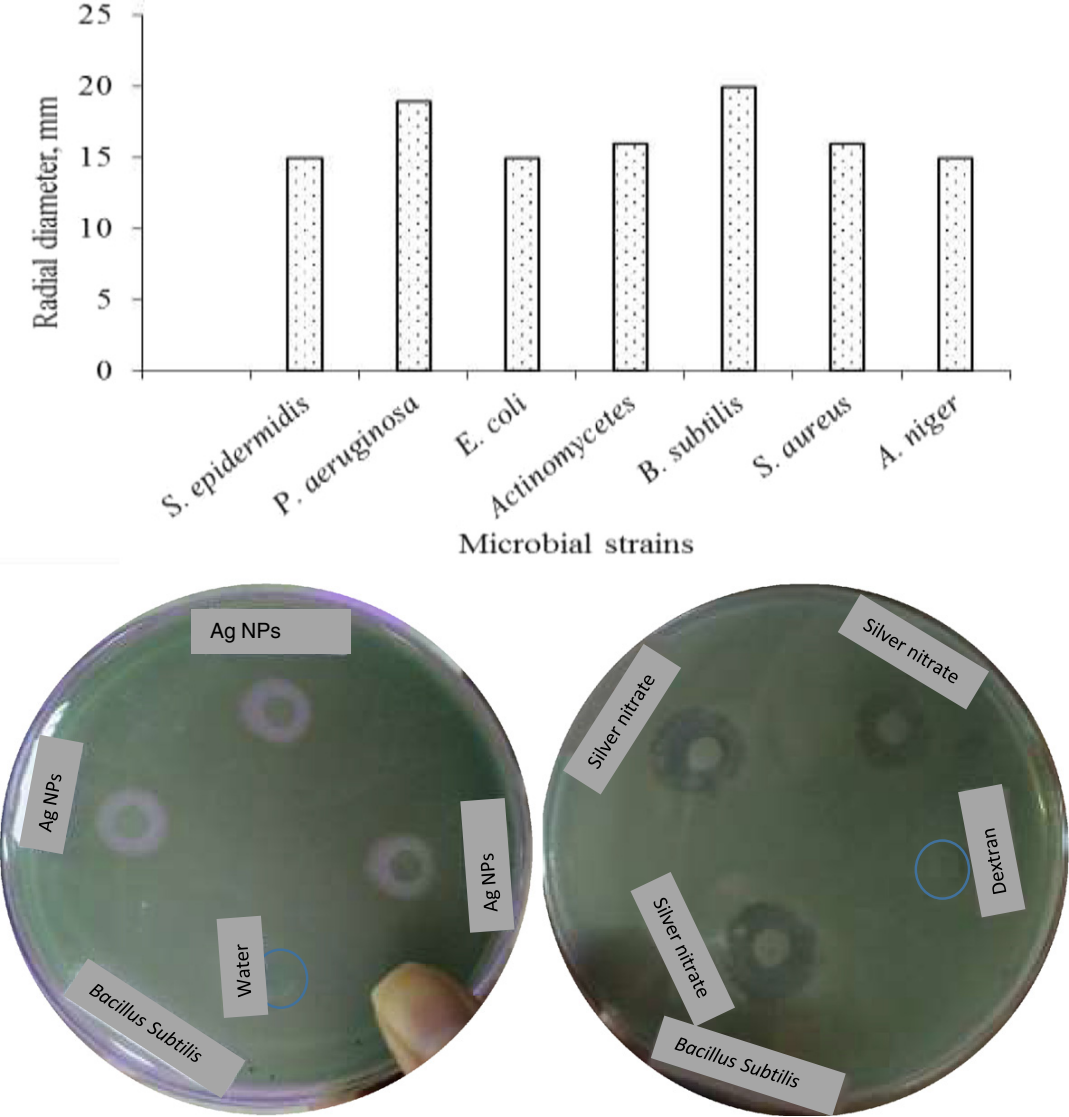
**Graph indicating inhibitory zone (radial diameter) of Ag NPs (50** ***mmol*** **)** ***vs*** **. different microbial strains whereas plates indicating that deionized water and dextran did not show any activity however, AgNO**_**3**_ **solution (0.01 M) was found active against** ***Bacillus subtilis*** **strains.**

Solution of Ag NPs showed significant antimicrobial activity against different bacterial (*S. aureus*, *E. coli, B. subtilis, S. epidermidis, P. aeruginosa)* and fungal strains *Actinomycetes and A. niger* as depicted in Figure 7. The inhibiting zones vs. microbial strains for Ag NPs solution of 50 mmol concentration and antibacterial activity of silver nanoparticles (Ag NPs) against *Bacillus subtilis* are also shown as a typical example (see Figure 7). It was observed that deionized water and dextran do not show any activity however, AgNO_3_ solution (0.01 M) was found active against mentioned strains. All of the experiments were carried out in triplicate and mean values have been reported. The prepared pristine Ag NPs can be used as effective therapeutic tools.

## Conclusions

We report on the diffused sun light assisted green synthesis of dextran stabilized Ag NPs without use of any hazardous and costly reducing agent or any extra functionalizing ligand. The as synthesized nanoparticles can be stored in solid state over months without imparting any change in the physical or optical properties. Being significantly active against microbes, the Ag NPs can be exploited for antimicrobial medical devices.
